# Multicollinearity and redundancy of the PET radiomic feature set

**DOI:** 10.1007/s00330-025-11637-7

**Published:** 2025-05-07

**Authors:** Wyanne A. Noortman, Dennis Vriens, Johan Bussink, Tineke W. H. Meijer, Erik H. J. G. Aarntzen, Christophe M. Deroose, Renaud Lhommel, Nicolas Aide, Christophe Le Tourneau, Elizabeth J. de Koster, Wim J. G. Oyen, Lianne Triemstra, Jelle P. Ruurda, Erik Vegt, Lioe-Fee de Geus-Oei, Floris H. P. van Velden

**Affiliations:** 1 Department of Radiology, Section of Nuclear Medicine, Leiden University Medical Center, Leiden, The Netherlands; 2 Department of Medical Oncology, University Medical Center Groningen, Groningen, The Netherlands; 3 Department of Medical Imaging, Radboud University Medical Center, Nijmegen, The Netherlands; 4 Department of Radiation Oncology, Radboud University Medical Center, Nijmegen, The Netherlands; 5 Department of Radiation Oncology, University Medical Center Groningen, Groningen, The Netherlands; 6 Department of Nuclear Medicine and Molecular Imaging, University Medical Center Groningen, Groningen, The Netherlands; 7 Department of Nuclear Medicine, Eberhard Karls University, Tuebingen, Germany; 8 Nuclear Medicine, UZ Leuven, Leuven, Belgium; 9 Division of Nuclear Medicine and Institut de Recherche Clinique, Cliniques Universitaires Saint Luc (UCLouvain), Brussels, Belgium; 10 INSERM ANTICIPE U1086, François Baclesse Cancer Centre, Caen, France; 11 Department of Drug Development and Innovation, Institut Curie, Paris-Saclay University, Paris, France; 12 Department of Radiology and Nuclear Medicine, Rijnstate Hospital, Arnhem, The Netherlands; 13 Department of Biomedical Sciences and Humanitas Clinical and Research Centre, Department of Nuclear Medicine, Humanitas University, Milan, Italy; 14 Department of Surgery, University Medical Center Utrecht, Utrecht, The Netherlands; 15 Department of Radiology and Nuclear Medicine, Erasmus MC University Medical Center Rotterdam, Rotterdam, The Netherlands; 16 Biomedical Photonic Imaging Group, University of Twente, Enschede, The Netherlands

**Keywords:** Radiomics, PET-CT, Malignancies, Multicollinearity, Feature reduction

## Abstract

**Introduction:**

The aim of this study was to map multicollinearity of the radiomic feature set in five independent [^18^F]FDG-PET cohorts with different tumour types and identify generalizable non-redundant features.

**Methods:**

Five [^18^F]FDG-PET radiomic cohorts were analysed: non-small cell lung carcinomas (*N* = 35), pheochromocytomas and paragangliomas (*N* = 40), head and neck squamous cell carcinomas (*N* = 54), [^18^F]FDG-positive thyroid nodules with indeterminate cytology (*N* = 84), and gastric carcinomas (*N* = 206). Lesions were delineated, and 105 radiomic features were extracted using PyRradiomics. In every cohort, Spearman’s rank correlation coefficient (ρ) matrices of features were calculated to determine which features showed (very) strong (ρ > 0.7 and ρ > 0.9) correlations with any other feature in all five cohorts. Cluster analysis of an averaged correlation matrix for all cohorts was performed at a threshold of ρ = 0.7 and ρ = 0.9. For each cluster, a representative, non-redundant feature was selected.

**Results:**

Seventy-two and 90 out of 105 features showed a (very) strong correlation with another feature in the correlation matrix in all five cohorts. Cluster analysis resulted in 35 and 15 non-redundant features at thresholds of ρ = 0.9 and ρ = 0.7, including 6 and 3 shape features, 4 and 2 intensity features, and 25 and 10 texture features, respectively. Seventy or 90 redundant features could be omitted at these thresholds, respectively.

**Conclusion:**

At least two-thirds of the radiomic feature set could be omitted because of strong multicollinearity in multiple independent cohorts. More redundant features could be identified using a less conservative threshold. Future research should indicate whether multicollinearity of the radiomic feature set is similar for other radiopharmaceuticals and imaging modalities.

**Key Points:**

***Question*** *Radiomic feature sets contain many strongly correlating features, which results in statistical challenges*.

***Findings*** *Analysis of the correlation matrices showed that the same radiomic features were strongly correlated in five independent [*^*18*^*F]FDG-PET cohorts with different tumour types*.

***Clinical relevance*** *At least two-thirds of the radiomic feature set could be omitted, because of strong multicollinearity. More redundant features could be identified using a less conservative threshold*.

**Graphical Abstract:**

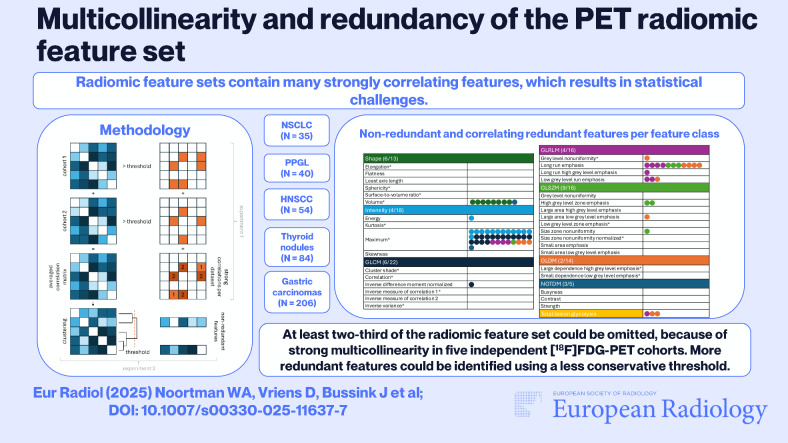

## Introduction

Since the first publication in 2009 [1], handcrafted radiomics has been investigated extensively to discover potential imaging biomarkers for quantification or monitoring of disease characteristics in clinical practice [2]. In the first years, many predefined, mathematical features from other image quantification fields have been utilised, and new features have been developed. This resulted in a standard radiomic feature set of approximately 100 features, but many variations exist [3]. These features range from straightforward and explainable intensity and shape-based features to more abstract texture features.

It is currently unclear what the radiomic feature set captures and how this is associated with tissue biology, as many of these handcrafted radiomic features lack a biological rationale. This becomes even more challenging when several radiomic features are combined into a radiomic signature. It is considered good radiomic practice to provide insight in the semantics or biological rationale of the selected features [4, 5]. However, earlier radiomic papers often lack this insight. For example, an early radiomic paper presented a radiomic signature consisting of four radiomic features that was associated with overall survival [6], but in later work, it was discovered that three of the four features in this signature strongly correlated with the tumour volume, showing similar model performances for the radiomic signature and tumour volume alone [7]. The radiomic feature set contains more strongly intercorrelating features: obvious correlations, such as between the maximum intensity and the mean intensity, but also less obvious correlations between abstract texture features without any demonstrated biological meaning.

This multicollinearity of the radiomic feature set results in statistical challenges. Overfitting of statistical models occurs when several strongly correlated features are introduced in a model [8]. In addition, machine learning models using cross-validation are impacted by multicollinearity, as multiple (non-overlapping or partly overlapping) sets of features are returned in supervised as well as unsupervised feature selection. This complicates the interpretation of the radiomic signature. The interpretability of radiomic signatures would also be improved by a smaller number of features, so that researchers know the mathematical definition of all individual features. Lastly, sample size calculations for radiomic studies are practically infeasible, since it is not possible to create in silico radiomic data without insight into multicollinearity.

It is well known that the radiomic feature set is multicollinear, but its nature and extensiveness are not fully understood. It is largely unknown which features are strongly correlated, but it is hypothesised that multicollinearity is, to some extent, similar between cohorts, even though this might vary as a result of disease setting and imaging modality, including the radiopharmaceutical used. If multicollinearity is similar between cohorts, redundant features could be removed, reducing statistical challenges.

The aim of this study was to map multicollinearity of the radiomic feature set by analysing similarities in correlations of radiomic feature sets in five independent 2-[^18^F]fluoro-2-deoxy-d-glucose ([^18^F]FDG) positron emission tomography (PET) cohorts, each of patients with a different tumour type. In this way, redundant features were identified and recommendations for a non-redundant feature set were provided. A smaller, non-redundant feature set could improve the interpretability and interoperability of radiomic signatures.

## Materials and methods

### Data

Data of five previously published [^18^F]FDG PET radiomic studies were retrospectively analysed: a cohort of 35 patients with non-small cell lung carcinomas (NSCLC), a cohort of 40 patients with pheochromocytomas and paragangliomas (PPGL), a cohort of 54 patient with head and neck squamous cell carcinomas (HNSCC), a cohort of 84 patients with [^18^F]FDG-positive thyroid nodules with indeterminate cytology (i.e. Bethesda 3 and 4), and a cohort of 206 patients with gastric carcinomas [9–13]. In the HNSCC cohort, only the baseline scans were used for analysis. These cohorts were selected, because they reflected different tumour biology and a different range of standardised uptake values (SUV) and metabolic tumour volumes (MTV). Some radiomic signatures could classify or predict clinical outcome, while others did not, and radiomic features were already extracted.

### Quantitative image analysis

Quantitative image analysis settings varied slightly between cohorts (Table 1). Volumes of interest (VOIs) were delineated on lesions using an adaptive threshold or using a fuzzy locally adaptive Bayesian (FLAB) algorithm [14, 15]. An adaptive threshold of 41% and 50% of SUV_peak_ was applied, obtained using a sphere of 12 mm diameter [16], corrected for local background, using in-house developed software or using the Accurate tool (AmsterdamUMC) [17]. Boxing was applied to exclude surrounding [^18^F]FDG-avid tissue. For FLAB, [^18^F]FDG-avid non-tumour tissue was excluded by drawing an oversized container around the tumour. The adaptive threshold was selected based on the range of SUVs in the dataset.

**Table 1:** Settings of quantitative image analysis for the different cohorts

	NSCLC	PPGL	HNSCC	Thyroid nodules with indeterminate cytology	Gastric carcinomas
Number of patients	35	40	54	84	206
Delineation method	FLAB^*^	Adaptive threshold 41%^**^	Adaptive threshold 50%	Adaptive threshold 50%	Adaptive threshold 50%
Bin width (g/mL)	0.55	0.5	0.5	0.5	0.5
Interpolated voxel size (mm³)	3.38 × 3.38 × 3.38	3.18 × 3.18 × 3.00	4.00 × 4.00 × 4.00	4.00 × 4.00 × 4.00	4.00 × 4.00 × 4.00
Interpolation method	Trilinear, grids aligned by centre	Not interpolated	B-spline, grids aligned by input origin	B-spline, grids aligned by input origin	B-spline, grids aligned by input origin
Texture parameters	Texture matrix aggregation: 3D (average) Distance weighting: no weighting Cooccurrence matrix symmetry: symmetric Linkage distance: Chebyshev distance of 1 Dependence matrix coarseness: 0				
Pyradiomics version	2.0	3.0	3.0	2.1.2	3.0
SUV_max_ (g/mL, median (range))	13.6 (5.6–31.0)	4.8 (1.7–36.0)	11.1 (4.8–25.2)	6.7 (2.0–68.1)	6.9 (1.5–51.4)
MTV (mL, median (range))	33.6 (7.9–180.9)	29.1 (2.3–253.9)	5.7 (1.6–42.2)	8.3 (1.7–115.6)	17.8 (2.6–135.0)
Original publication	[9]	[10]	[11]	[12]	[13]

*NSCLC* non-small cell lung carcinomas, *PPGL* pheochromocytomas and paragangliomas, *HNSCC* head and neck squamous cell carcinomas, *FLAB* fuzzy locally adaptive Bayesian, *SUV*_max_ maximum standardised uptake value, *MTV* metabolic tumour volume

^*^ FLAB segmentation is a suitable method for the delineation of lesions with heterogeneous activity concentrations

^**^ As PPGLs are characterised by low [^18^F]FDG uptake, a lower threshold was found appropriate

Radiomic feature extraction was performed in PyRadiomics version 2.0, 2.1.2, or 3.0 [18]. All PyRadiomic versions complied with the Image Biomarker Standardisation Initiative (IBSI) [3]. For all VOIs, 104 radiomic features were extracted: 13 shape features, 18 first-order features, and 73 texture features, including 22 grey level cooccurrence matrix (GLCM), 16 grey level run length matrix (GLRLM), 16 grey level size zone matrix (GLSZM), 14 grey level dependence matrix (GLDM), and 5 neighbouring grey tone difference matrix (NGTDM) features. In addition, the total lesion glycolysis (TLG), the product of the mean SUV and the MTV, was calculated. A fixed bin size of 0.5 g/mL or 0.55 g/mL was applied. (Interpolated) Voxel sizes were standardised per cohort and ranged from 3.18 × 3.18 × 3.00 mm³ to 4.00 × 4.00 × 4.00 mm³. Please refer to the original publications for specific details on image acquisition, reconstruction, and data analysis [9–13].

### Statistical analysis

**Fig. 1:**
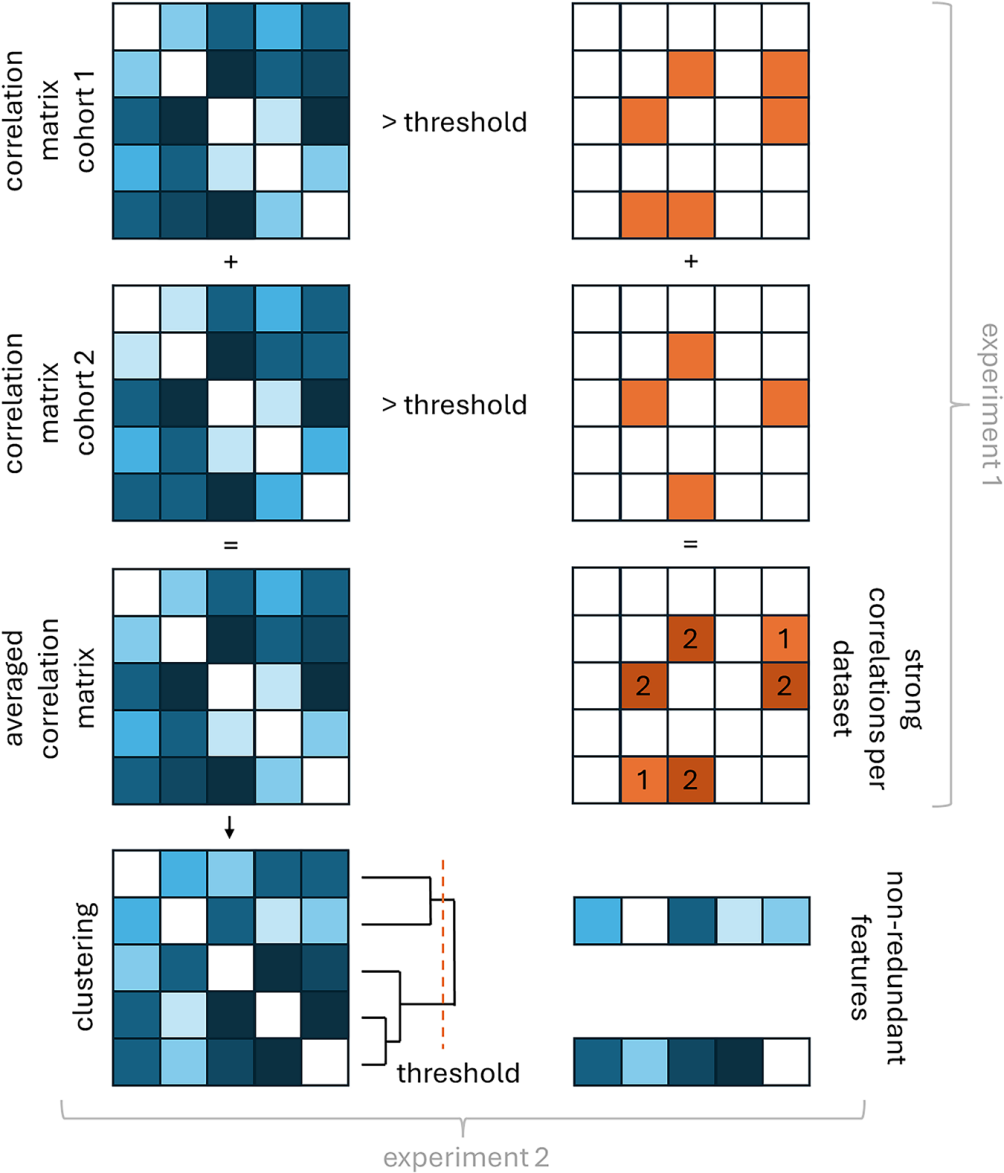
Experiments to assess multicollinearity and redundancy of radiomic features in five clinical cohorts based on the absolute Spearman correlation matrices. In experiment 1, it was determined which features showed (very) strong correlations with other features in all five cohorts or in four of the five cohorts, indicating redundancy. In experiment 2, an averaged correlation matrix of all cohorts was computed, and cluster analysis was performed to group (very) strongly correlating features. From each cluster, a representative non-redundant feature was selected, selecting the feature with the highest summed correlation with all other features in the cluster

For all cohorts, absolute Spearman’s rank correlation coefficient (ρ) matrices of radiomic features were calculated in MATLAB 2024a (Mathworks). Multicollinearity and redundancy were assessed in 2 experiments (Fig. 1). In experiment 1, it was determined in each cohort which features showed (very) strong (ρ > 0.7 or ρ > 0.9) correlations with other features. Subsequently, it was determined which features showed a (very) strong correlation with another feature in each cohort, in all five cohorts or in four of the five cohorts, indicating redundancy. A list of features that did not show a correlation ρ > 0.7 with any other feature was provided.

In experiment 2, the absolute correlation matrices for the different cohorts were averaged into one correlation matrix. Hierarchical clustering was performed to group (very) strongly correlating features (ρ > 0.7 or ρ > 0.9) based on single linkage and Euclidean distances. From each cluster a representative feature was selected, selecting the feature with the highest summed correlation with all other features in the cluster, unless the cluster contained a traditional quantitative PET feature (i.e. SUV_max_, SUV_mean_, metabolic tumour volume, and TLG), as these could often be computed in the software packages used in clinical practice.

## Results

**Fig. 2:**
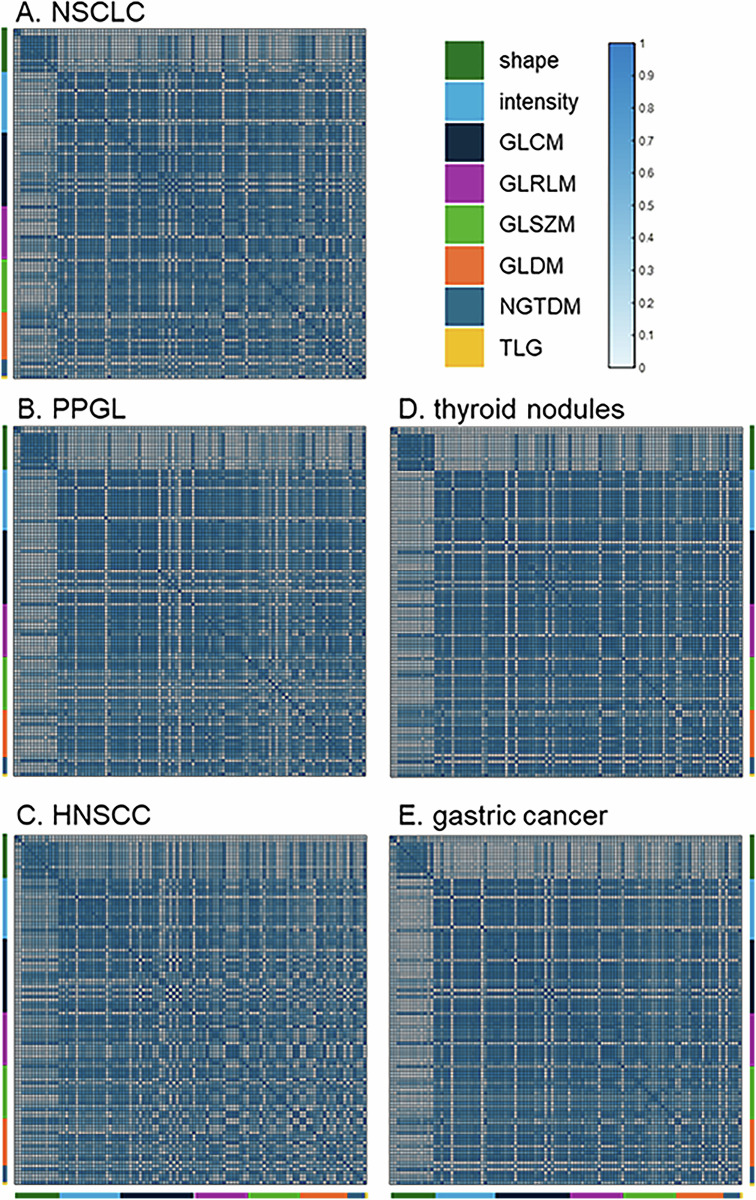
Absolute correlation matrices of radiomic features showing similar patterns in cohorts of (**A**) NSCLC, (**B**) PPGL, (**C**) HNSCC, (**D**) thyroid nodules, and (**E**) gastric cancer. NSCLC, non-small cell lung carcinomas; PPGL, pheochromocytomas and paragangliomas; HNSCC, head and neck squamous cell carcinomas; GLCM, grey level cooccurrence matrix; GLRLM, grey level run length matrix; GLSZM, grey level size zone matrix; GLDM, grey level dependence matrix; NGTDM, neighbouring grey tone difference matrix; TLG, total lesion glycolysis

Multicollinearity of the radiomic feature set was observed in all five cohorts, demonstrating similar patterns in the correlation matrices (Fig. 2). Seventy-two out of 105 features showed a very strong correlation (ρ > 0.9) with any other feature in all five cohorts; in the individual cohorts these numbers ranged from 85 to 92 (Table 2). This number increased to 83 when any four cohorts were taken into account. Even 90 out of 105 features showed a strong correlation (ρ > 0.7) with any other feature in all five cohorts, increasing to 101 when any four cohorts were considered.

**Table 2:** Number of features with a (very) strong correlation with any other feature in the feature set and a list of features that did not show a correlation ρ > 0.7 with any other feature per cohort, for all five datasets, and for any four out of five datasets

	Number of features with a very strong correlation (ρ > 0.9) with any other feature	Number of features with a strong correlation (ρ > 0.7) with any other feature	Features that did not show a correlation ρ > 0.7 with any other feature
NSCLC (*N* = 35)	92	103	- Sphericity (shape) - Small dependence, low grey level emphasis (GLDM)
PPGL (*N* = 40)	85	101	- Inverse measure of correlation 1 (GLCM) - Inverse variance (GLCM) - Size zone nonuniformity normalised (GLSZM) - Small area low grey level emphasis (GLSZM)
HNSCC (*N* = 54)	91	102	- Elongation (shape) - Flatness (shape) - Cluster shade (GLCM)
Thyroid nodules (*N* = 84)	91	101	- Kurtosis (intensity) - Cluster shade (GLCM) - Small area low grey level emphasis (GLSZM) - Small dependence, low grey level emphasis (GLDM)
Gastric carcinomas (*N* = 206)	88	102	- Surface to volume ratio (shape) - Correlation (GLCM) - Small dependence, low grey level emphasis (GLDM)
All five datasets	72	90	- Elongation (shape) - Flatness (shape) - Sphericity (shape) - Surface to volume ratio (shape) - Kurtosis (intensity) - Skewness (intensity) - Cluster shade (GLCM) - Correlation (GLCM) - Inverse measure of correlation 1 (GLCM) - Inverse measure of correlation 2 (GLCM) - Inverse variance (GLCM) - Size zone nonuniformity normalised (GLSZM) - Small area emphasis (GLSZM) - Small area low grey level emphasis (GLSZM) - Small dependence, low grey level emphasis (GLDM)
Any four out of five datasets	83	101	- Cluster shade (GLCM) - Correlation (GLCM) - Small area low grey level emphasis (GLSZM) - Small dependence, low grey level emphasis (GLDM)

*NSCLC* non-small cell lung carcinomas, *PPGL* pheochromocytomas and paragangliomas, *HNSCC* head and neck squamous cell carcinomas, *GLDM* grey level dependence matrix, *GLCM* grey level cooccurrence matrix, *GLSZM* grey level size zone matrix

Subsequently, cluster analysis of the averaged correlation matrix for all five cohorts identified 35 clusters of strongly correlating features (ρ > 0.9, Table 3). For each cluster, one representative feature was selected (Supplementary Table 1). The largest cluster contained 38 strongly correlating features; SUV_max_ being the representative feature of this cluster. This cluster contained features from all categories, including 13 out of 18 intensity features and 15 out of 22 GLCM features. Cluster analysis identified fifteen clusters of strongly correlating features (ρ > 0.7, Supplementary Table 2).

**Table 3:**
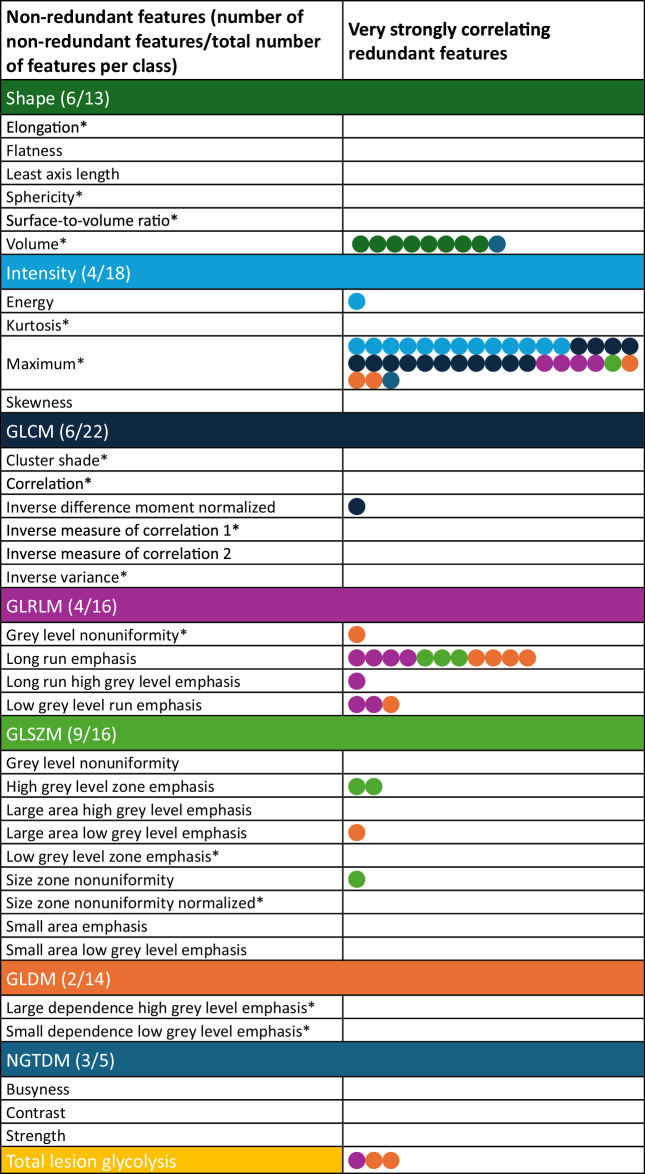
Representative non-redundant features and the number of very strongly correlating (ρ = 0.9) redundant features per feature class

Each dot in the right column represents one redundant feature, and the colour represents the feature class, which corresponds to the colours of the feature class headers. The names of the features in the column are presented in Supplementary Table 1. The asterisk (*) marks non-redundant features at a threshold of ρ = 0.7

## Discussion

In this study, multicollinearity of the radiomic feature set was assessed in five independent [^18^F]FDG PET cohorts in different tumour types, aiming at the identification of non-redundant features. We identified 72 radiomic features that showed a very strong correlation with any other radiomic feature in the feature set in all five cohorts. Cluster analysis resulted in a reduced feature set of 35 non-redundant features. When opting for a more conservative approach, considering strong (ρ > 0.7) correlations, the feature set could even be reduced to 15 features.

Multicollinearity is a well-known phenomenon within radiomic analysis that causes statistical challenges, but its nature and extent are not fully understood. In contrast to other -omics fields, radiomic features lack biological rationale. Individual genes, as studied in genomics, have a biological meaning, and associations between individual genes may provide insights into biological mechanisms, in patients, as well as in healthy subjects. In radiomics, on the other hand, features are predefined, and correlations between features do not contribute to additional insights into disease mechanisms. Large numbers of features, originating from different fields of image analysis, are extracted from images all at once, presuming that at least some of these features show associations with clinical outcome. However, as these features are strongly correlated, many features may be omitted without losing comprehension of the reflected underlying biology.

Many radiomic studies already account for multicollinearity by incorporating some form of redundancy filtering in the analysis, in some cases before feature selection [19], in others after feature selection and before introducing features into a classification or prediction model [20]. Other studies employ supervised feature selection methods that incorporate the removal of redundant features, such as minimum redundancy maximum relevance algorithms [21]. Several studies use regularised classifiers like LASSO (least absolute shrinkage and selection operator) regression for feature selection without redundancy filtering. However, these regularised classifiers still require redundancy filtering, since these classifiers do not have a unique minimiser in the case of high multicollinearity (like in radiomics) and their selection capability is constrained by the sample size, which may result in unstable prediction [22]. Some studies remove features that strongly correlate with conventional metrics like SUV_max_ or MTV, which could be an effective method, considering that SUV_max_ in the current study strongly correlates with about one-third of all features in the feature set. However, strong correlations between other features are not considered using this approach.

To the best of our knowledge, there are no studies that have investigated the similarities of redundant features in different cohorts to reduce the radiomic feature set. The feature set could also be reduced by selecting only reproducible features. Many studies have assessed the robustness and reproducibility of radiomic features related to factors associated with image acquisition, reconstruction, segmentation, and radiomics-specific processing steps [23, 24]. In general, shape and intensity features are more robust compared to texture features. The optimal radiomic feature set contains features that are both non-redundant and reproducible.

In the current study, two-thirds of radiomic features could be omitted due to redundancy, resulting in a radiomic feature set of 35 or fewer non-redundant features. The correlations between specific features may result from similarities in the mathematical definitions of features. In some cases, these definitions are quite similar, as features belong to the same feature class, but in other cases, similarities might be less obvious. The feature classes shape and NGTDM relatively contained the most non-redundant features, and these features were generally not related to features from other classes, suggesting different information than captured by other features. The GLSZM class included more non-redundant features than the GLRLM class, a possible explanation being the 3D configuration of the GLSZM features compared to the GLRLM features, capturing more information per feature. The feature classes GLCM and GLDM consisted of relatively few non-redundant features. However, it should be taken into account that the non-redundant features that were selected did not show high correlations with other features in the feature set, indicating different information. GLCM features were often correlated with intensity features, which also showed high redundancy. The intensity feature class consists of several features that represent a value in the intensity histogram; one metric being increased often suggests the other metric being increased as well.

In addition, the intensity histogram feature entropy is an often-reported feature in radiomic analysis and, moreover, reportedly is one of the most stable radiomic features [24]. However, we showed that entropy is very strongly correlated with SUV_max_ in all five clinical cohorts, indicating redundancy. Entropy is described as the complexity or randomness of pixel intensities in an image based on normalised histogram counts, defined as a function of the number of grey levels and the probability of a pixel having a certain grey level. Consequently, a higher number of grey levels, as indicated by a higher SUV_max_, tends to result in a higher value for entropy, compared to a smaller number of grey levels.

Reducing the number of radiomic features to a maximum of 35 features considerably reduces the statistical burden of overfitting and the difficulties related to interpretation of the radiomic signatures and sample size calculations. Multicollinearity of the input variables has the inherent risk of overfitting the classification or prediction model. A regression model represents the mean change in a dependent variable for each unit of change in an independent variable, when all other independent variables are held constant. In case of strong correlations between independent variables, changes in one are associated with changes in the other variable. Accordingly, the regression coefficients can fluctuate widely based on the other independent variables in the model and become sensitive to small changes, resulting in overfitting of the model.

In addition, feature selection may seem trivial, but it is a significant source of variation in many radiomic studies [25]. Feature selection in a multicollinear dataset could be challenging, for instance, as it may return multiple (correlating) sets of features in the different folds of cross-validation, complicating the interpretation of the radiomic signature. With only 35 features, there is still a need for feature selection, but the process is more manageable.

Furthermore, a non-redundant feature set may pave the way towards sample size calculations for radiomic studies, which may help to assess the feasibility of a labour-intensive radiomic study. Most radiomic researchers utilise a minimum sample size ranging between 80 and 200 patients for handcrafted radiomics, which may be sufficient as a proof-of-concept study in case of a well-reflected biological signal and when cross-validation and sham experiments are performed. With a non-redundant feature set and insight into the distribution of the radiomic features, sample size calculations could be performed for a specific algorithm.

Our study has several strengths and limitations. A strength is that a smaller radiomic feature set simplifies the radiomic analysis, as the analysis no longer requires correlation-based redundancy filtering. Another strength is that the multicollinearity of the feature set was compared in five independent cohorts, all demonstrating similar patterns of multicollinearity, which indicates the robustness of our findings. As we retrospectively analysed these five cohorts, the settings of feature extraction vary slightly between cohorts, which is both a strength and a limitation. Homogeneous preprocessing settings would have substantiated our findings. Nevertheless, we were able to demonstrate that redundancy is similar between cohorts, independent of the preprocessing settings. We may even conclude that these small variations in preprocessing settings did not significantly influence the redundancy of features, which is underlined by findings by Marzi et al [26]. They assessed the effect of voxel size resampling, discretisation, and filtering on correlation-based dimensionality reduction in radiomic features from cardiac T1 and T2 maps, demonstrating that voxel size and discretisation showed a high stability index, indicating that these settings did not significantly impact redundancy filtering.

Similarly, our findings are also independent of batch effects introduced by the use of different scanners. Redundancy is similar between the PPGL cohort, of which all PET scans were acquired with the same scanner, and the other cohorts, in which different scanners were used. These results suggest that the observed redundancy is inherent to the mathematical definitions of the features, instead of the biological properties of the tumours and the imaging physics. However, more research is warranted to verify whether a similar redundancy could be observed in other imaging modalities. Another limitation is that we did not consider the *p*-values of the Spearman correlations. These *p*-values could vary widely, as a result of sample sizes of the individual cohorts ranging from 35 to 206. It was observed that the features showing (very) strong correlations were practically the same in all five datasets. In addition, it should be noted that the cluster analysis was performed based on an averaged correlation matrix for all five cohorts, thereby balancing correlations for individual cohorts. With correlations between specific features in four of the five cohorts of 0.99, the correlation in the fifth cohort could theoretically be as low as 0.6, while still resulting in a very strong average correlation above 0.9.

Furthermore, a different number of non-redundant features would have been selected at a different threshold. Also, we only analysed a subset of 105 IBSI-compliant features. In a feature set with additional features, some features may correlate strongly with one of the identified clusters, while others may be non-redundant. Along these lines, our analysis only applies to handcrafted radiomics, as deep learning radiomics does not employ predefined features, but features are developed based on the input images. In addition, future research should indicate whether multicollinearity of the radiomic feature set is similar in other disease settings, for other radiopharmaceuticals in PET, and in other imaging modalities. Lastly, it may be interesting to test the performance of a radiomic model with only non-redundant features compared to the model with all features.

In conclusion, the radiomic feature set contains many redundant features. At least two-thirds of the radiomic feature set could be omitted, because of the strong multicollinearity of the features in five independent clinical [^18^F]FDG PET cohorts. More redundant features could be identified using a less conservative threshold.

## Supplementary information

ELECTRONIC SUPPLEMENTARY MATERIAL

## Abbreviations

[^18^F]FDG: 2-[^18^F]fluoro-2-deoxy-d-glucose
FLAB: Fuzzy locally adaptive Bayesian
GLCM: Grey level cooccurrence matrix
GLDM: Grey level dependence matrix
GLRLM: Grey level run length matrix
GLSZM: Grey level size zone matrix
HNSCC: Head and neck squamous cell carcinomas
IBSI: Image biomarker standardisation inititiative
MTV: Metabolic tumour volume
NGTDM: Neighbouring grey tone difference matrix
NSCLC: Non-small cell lung carcinomas
PET: Positron emission tomography
PPGL: Pheochromocytomas and paragangliomas
SUV: Standardised uptake value
TLG: Total lesion glycolysis
VOI: Volume of interest

## References

[1] El Naqa I, Grigsby P, Apte A et al (2009) Exploring feature-based approaches in PET images for predicting cancer treatment outcomes. Pattern Recognit 42:1162–1171 20161266 10.1016/j.patcog.2008.08.011 PMC2701316

[2] Limkin EJ, Sun R, Dercle L et al (2017) Promises and challenges for the implementation of computational medical imaging (radiomics) in oncology. Ann Oncol 28:1191–1206 28168275 10.1093/annonc/mdx034

[3] Zwanenburg A, Vallières M, Abdalah MA et al (2020) The image biomarker standardization initiative: standardized quantitative radiomics for high-throughput image-based phenotyping. Radiology 295:328–338 32154773 10.1148/radiol.2020191145 PMC7193906

[4] Tomaszewski MR, Gillies RJ (2021) The biological meaning of radiomic features. Radiology 298:505–516 33399513 10.1148/radiol.2021202553 PMC7924519

[5] Yip SSF, Liu Y, Parmar C et al (2017) Associations between radiologist-defined semantic and automatically computed radiomic features in non-small cell lung cancer. Sci Rep 7:3519 28615677 10.1038/s41598-017-02425-5 PMC5471260

[6] Aerts HJWL, Velazquez ER, Leijenaar RTH et al (2014) Decoding tumour phenotype by noninvasive imaging using a quantitative radiomics approach. Nat Commun 5:4006 24892406 10.1038/ncomms5006 PMC4059926

[7] Welch ML, McIntosh C, Haibe-Kains B et al (2019) Vulnerabilities of radiomic signature development: The need for safeguards. Radiother Oncol 130:2–9 30416044 10.1016/j.radonc.2018.10.027

[8] Clarke R, Ressom HW, Wang A et al (2008) The properties of high-dimensional data spaces: implications for exploring gene and protein expression data. Nat Rev Cancer 8:37–49 18097463 10.1038/nrc2294 PMC2238676

[9] Noortman WA, Vriens D, Slump CH et al (2020) Adding the temporal domain to PET radiomic features. PLoS One 15:e0239438 32966313 10.1371/journal.pone.0239438 PMC7510999

[10] Noortman WA, Vriens D, de Geus-Oei L-F et al (2022) [^18^F]FDG-PET/CT radiomics for the identification of genetic clusters in pheochromocytomas and paragangliomas. Eur Radiol 32:7227–7236 36001126 10.1007/s00330-022-09034-5 PMC9474528

[11] Noortman WA, Aide N, Vriens D et al (2023) Development and external validation of a PET radiomic model for prognostication of head and neck Cancers (Basel) 15:2681 10.3390/cancers15102681 PMC10216021 37345017

[12] de Koster EJ, Noortman WA, Mostert JM et al (2022) Quantitative classification and radiomics of [(18)F]FDG-PET/CT in indeterminate thyroid nodules. Eur J Nucl Med Mol Imaging 49:2174–2188 35138444 10.1007/s00259-022-05712-0 PMC9165273

[13] Pullen LCE, Noortman WA, Triemstra L et al (2023) Prognostic value of [(18)F]FDG PET radiomics to detect peritoneal and distant metastases in locally advanced gastric cancer—a side study of the prospective multicentre PLASTIC study. Cancers (Basel) 15:2874 10.3390/cancers15112874 PMC10251816 37296837

[14] Hatt M, Cheze le Rest C, Turzo A, Roux C, Visvikis D (2009) A fuzzy locally adaptive Bayesian segmentation approach for volume determination in PET. IEEE Trans Med Imaging 28:881–893 19150782 10.1109/TMI.2008.2012036 PMC2912931

[15] Frings V, van Velden FH, Velasquez LM et al (2014) Repeatability of metabolically active tumor volume measurements with FDG PET/CT in advanced gastrointestinal malignancies: a multicenter study. Radiology 273:539–548 24865311 10.1148/radiol.14132807

[16] Wahl RL, Jacene H, Kasamon Y, Lodge MA (2009) From RECIST to PERCIST: evolving considerations for PET response criteria in solid tumors. J Nucl Med 50:122s–150s 19403881 10.2967/jnumed.108.057307 PMC2755245

[17] Boellaard R (2018) Quantitative oncology molecular analysis suite: ACCURATE. Soc Nuclear Med 59:1753

[18] Van Griethuysen JJ, Fedorov A, Parmar C et al (2017) Computational radiomics system to decode the radiographic phenotype. Cancer Res 77:e104–e107 29092951 10.1158/0008-5472.CAN-17-0339 PMC5672828

[19] Martens RM, Koopman T, Noij DP et al (2020) Predictive value of quantitative (18)F-FDG-PET radiomics analysis in patients with head and neck squamous cell carcinoma. EJNMMI Res 10:102 32894373 10.1186/s13550-020-00686-2 PMC7477048

[20] Dissaux G, Visvikis D, Da-ano R et al (2020) Pretreatment 18F-FDG PET/CT radiomics predict local recurrence in patients treated with stereotactic body radiotherapy for early-stage non–small cell lung cancer: a multicentric study. J Nucl Med 61:814–820 31732678 10.2967/jnumed.119.228106

[21] Xie Y, Zhao H, Guo Y et al (2021) A PET/CT nomogram incorporating SUVmax and CT radiomics for preoperative nodal staging in non-small cell lung cancer. Eur Radiol 31:6030–6038 33560457 10.1007/s00330-020-07624-9 PMC8270849

[22] Peeters CF, Übelhör C, Mes SW et al (2019) Stable prediction with radiomics data. Preprint at 10.48550/arXiv:190311696

[23] Zwanenburg A (2019) Radiomics in nuclear medicine: robustness, reproducibility, standardization, and how to avoid data analysis traps and replication crisis. Eur J Nucl Med Mol Imaging 46:2638–2655 31240330 10.1007/s00259-019-04391-8

[24] Traverso A, Wee L, Dekker A, Gillies R (2018) Repeatability and reproducibility of radiomic features: a systematic review. Int J Radiat Oncol Biol Phys 102:1143–1158 30170872 10.1016/j.ijrobp.2018.05.053 PMC6690209

[25] Parmar C, Grossmann P, Bussink J, Lambin P, Aerts H (2015) Machine learning methods for quantitative radiomic biomarkers. Sci Rep 5:13087 26278466 10.1038/srep13087 PMC4538374

[26] Marzi C, Marfisi D, Barucci A et al (2023) Collinearity and dimensionality reduction in radiomics: effect of preprocessing parameters in hypertrophic cardiomyopathy magnetic resonance T1 and T2 mapping. Bioengineering 10:80 10.3390/bioengineering10010080 PMC9854492 36671652

[27] Noortman WA, Vriens D, Mooij CDY et al (2021) The influence of the exclusion of central necrosis on [18F]FDG PET radiomic analysis. Diagnostics (Basel) 11:1296 10.3390/diagnostics11071296 PMC8304274 34359379

