# Supplementary material for: Multicollinearity and redundancy of the PET radiomic feature set
Source: Eur Radiol. 2025 May 7;35(11):6905–16. doi: 10.1007/s00330-025-11637-7 (PMC12559118; doi:10.1007/s00330-025-11637-7)
Supplement: Supplementary file 1 — ELECTRONIC SUPPLEMENTARY MATERIAL [file 330_2025_11637_MOESM1_ESM.pdf]

Multicollinearity and redundancy of the PET radiomic feature set

ELECTRONIC SUPPLEMENTARY MATERIAL

Eur Radiol (2025) Noortman WA, Vriens D, Bussink J, et al.

Supplementary table 1. Cluster analysis of averaged correlation matrix in five independent [^{18}F]FDG PET cohorts at a very strong Spearman correlation (r>0.9). Table presents representative non-redundant features, correlating redundant features, and an explanation of the cluster. GLCM: grey level cooccurrence matrix, GLRLM: grey level run length matrix, GLSZM: grey level size zone matrix, GLDM: grey level dependence matrix, NGTDM: neighbouring grey tone difference matrix, TLG: total lesion glycolysis.

Non-redundant feature (number non-redundant features/total number of features)	Very strongly correlating redundant features	Cluster explanation
**Shape (6/13)**		
Elongation		Ratio between longest and second largest axis
Flatness		Ratio between the longest and shortest axis
Least axis length		Shortest axis
Sphericity		Roundness
Surface-to-volume ratio		Ratio between area and volume
Volume	Major axis length (shape)<br>Maximum 2D diameter column (shape)<br>Maximum 2D diameter row (shape)<br>Maximum 2D diameter slice (shape)<br>Maximum 3D diameter (shape)<br>Minor axis length (shape)<br>Surface area (shape)<br>Coarseness (NGTDM)	Size
**Intensity (4/18)**		
Energy	Total energy (intensity)	Magnitude of voxel values, size-dependent
Kurtosis		'Peakedness' of the voxel value distribution
Maximum	10th Percentile (intensity)<br>90th Percentile (intensity)<br>Entropy (intensity)	Highest voxel intensity

	Interquartile range (intensity)<br>Mean (intensity)<br>Mean absolute deviation (intensity)<br>Median (intensity)<br>Minimum (intensity)<br>Range (intensity)<br>Robust mean absolute deviation (intensity)<br>Root mean squared (intensity)<br>Uniformity (intensity)<br>Variance (intensity)<br>Autocorrelation (GLCM)<br>Cluster prominence (GLCM)<br>Cluster tendency (GLCM)<br>Contrast (GLCM)<br>Difference average (GLCM)<br>Difference entropy (GLCM)<br>Difference variance (GLCM)<br>Inverse difference (GLCM)<br>Inverse difference moment (GLCM)<br>Joint average (GLCM)<br>Joint energy (GLCM)<br>Joint entropy (GLCM)<br>Maximum probability (GLCM)<br>Sum entropy (GLCM)<br>Sum squares (GLCM)<br>Grey level nonuniformity normalized (GLRLM)<br>Grey level variance (GLRLM)<br>High grey level run emphasis (GLRLM)<br>Short run high grey level emphasis (GLRLM)<br>Grey level variance (GLSZM)<br>Grey level variance (GLDM)<br>High grey level emphasis (GLDM)	

	Small dependence high grey level emphasis (GLDM)<br>Complexity (NGTDM)	
Skewness		Asymmetry of the voxel value distribution
**GLCM (6/22)**		
Cluster shade		Independency of voxel values between neighbouring voxels
Correlation		Dependency of voxel values between neighbouring voxels
Inverse difference moment normalized	Inverse difference normalized (GLCM)	Local homogeneity
Inverse measure of correlation 1		Complexity of the texture
Inverse measure of correlation 2		Complexity of the texture
Inverse variance		Local homogeneity
**GLRLM (4/16)**		
Grey level nonuniformity	Grey level nonuniformity (GLDM)	Similarity of voxel values
Long run emphasis	Run length nonuniformity normalized (GLRLM)<br>Run percentage (GLRLM)<br>Run variance (GLRLM)<br>Short run emphasis (GLRLM)<br>Large area emphasis (GLSZM)<br>Zone percentage (GLSZM)<br>Zone variance (GLZSM)<br>Dependence nonuniformity normalized (GLDM)<br>Dependence variance (GLDM)<br>Large dependence emphasis (GLDM)<br>Small dependence emphasis (GLDM)	Row of voxels with the same value
Long run high grey level emphasis	Run entropy (GLRLM)	Row of voxels with a high value
Low grey level run emphasis	Long run low Grey level emphasis (GLRLM)	Row of voxels with a low value

	Short run low Grey level emphasis (GLRLM)<br>Low grey level emphasis (GLDM)	
**GLSZM (9/16)**		
Grey level nonuniformity		Variability of voxel values
High grey level zone emphasis	Grey level nonuniformity normalized (GLSZM)<br>Small area high grey level emphasis (GLZSM)	Clusters of voxels with high value
Large area high grey level emphasis		Large clusters of voxels with high value
Large area low grey level emphasis	Large dependence low grey level emphasis (GLDM)	Clusters of voxels with low value
Low grey level zone emphasis		Clusters of voxels with low value
Size zone nonuniformity	Zone entropy (GLSZM)	Variability of cluster sizes
Size zone nonuniformity normalized		Variability of cluster sizes
Small area emphasis		Fine texture with small clusters
Small area low grey level emphasis		Small clusters with low value
**GLDM (2/14)**		
Large dependence high grey level emphasis		Large clusters of voxels with high values
Small dependence low grey level emphasis		Small clusters of voxels with low values
**NGTDM (3/5)**		
Busyness		Rapid changes in intensity between neighbouring voxels
Contrast		Changes in intensity between neighbouring voxels combined with the intensity range
Strength		Slow changes in intensity between neighbouring voxels

**Total lesion glycolysis**	Run length nonuniformity (GLRLM)<br>Dependence entropy (GLDM)<br>Dependence nonuniformity (GLDM)	Product of intensity and volume

Supplementary table 2. Cluster analysis of averaged correlation matrix in five independent [18F]FDG PET cohorts at a strong Spearman correlation (r>0.7). Table presents representative non-redundant features, correlating redundant features, and an explanation of the cluster. GLCM: grey level cooccurrence matrix, GLRLM: grey level run length matrix, GLSZM: grey level size zone matrix, GLDM: grey level dependence matrix, NGTDM: neighbouring grey tone difference matrix, TLG: total lesion glycolysis.

Non-redundant feature (number non-redundant features/total number of features)	Strongly correlating redundant features	Cluster explanation
**Shape (4/13)**		
Elongation	Flatness (shape)	Ratio between the axes
Sphericity		Roundness
Surface-to-volume ratio		Ratio between area and volume
Volume	Least axis length (shape)<br>Major axis length (shape)<br>Maximum 2D diameter column (shape)<br>Maximum 2D diameter row (shape)<br>Maximum 2D diameter slice (shape)<br>Maximum 3D diameter (shape)<br>Minor axis length (shape)<br>Surface area (shape)<br>Energy (intensity)<br>Total energy (intensity)<br>Total lesion glycolysis<br>Run length nonuniformity (GLRLM)<br>Grey level nonuniformity (GLSZM)<br>Dependence entropy (GLDM)<br>Dependence nonuniformity (GLDM)<br>Coarseness (NGTDM)	Size
**Intensity (2/18)**		
Kurtosis	Skewness (intensity)	Shape of the voxel value distribution

Maximum	10th Percentile (intensity)<br>90th Percentile (intensity)<br>Entropy (intensity)<br>Interquartile range (intensity)<br>Mean (intensity)<br>Mean absolute deviation (intensity)<br>Median (intensity)<br>Minimum (intensity)<br>Range (intensity)<br>Robust mean absolute deviation (intensity)<br>Root mean squared (intensity)<br>Uniformity (intensity)<br>Variance (intensity)<br>Autocorrelation (GLCM)<br>Cluster prominence (GLCM)<br>Cluster tendency (GLCM)<br>Contrast (GLCM)<br>Difference average (GLCM)<br>Difference entropy (GLCM)<br>Difference variance (GLCM)<br>Inverse difference (GLCM)<br>Inverse difference moment (GLCM)<br>Joint average (GLCM)<br>Joint energy (GLCM)<br>Joint entropy (GLCM)<br>Maximum probability (GLCM)<br>Sum entropy (GLCM)<br>Sum squares (GLCM)<br>Grey level nonuniformity normalized (GLRLM)<br>Grey level variance (GLRLM)<br>High grey level run emphasis (GLRLM)<br>Long run emphasis (GLRLM)<br>Long run high grey level emphasis (GLRLM)	Highest voxel intensity

	Long run low grey level emphasis (GLRLM)<br>Low grey level run emphasis (GLRLM)<br>Run entropy (GLRLM)<br>Run length nonuniformity normalized (GLRLM)<br>Run percentage (GLRLM)<br>Run variance (GLRLM)<br>Short run emphasis (GLRLM)<br>Short run high grey level emphasis (GLRLM)<br>Short run low grey level emphasis (GLRLM)<br>Grey level variance (GLSZM)<br>Grey level nonuniformity normalized (GLSZM)<br>High grey level zone emphasis (GLSZM)<br>Large area emphasis (GLSZM)<br>Size zone nonuniformity (GLSZM)<br>Small area high grey level emphasis (GLZSM)<br>Zone entropy (GLSZM)<br>Zone percentage (GLSZM)<br>Zone variance (GLZSM)<br>Dependence nonuniformity normalized (GLDM)<br>Dependence variance (GLDM)<br>Grey level variance (GLDM)<br>High grey level emphasis (GLDM)<br>Large area low grey level emphasis (GLSZM)<br>Large dependence emphasis (GLDM)<br>Large dependence low grey level emphasis (GLDM)	

	Low grey level emphasis (GLDM)<br>Small dependence emphasis (GLDM)<br>Small dependence high grey level emphasis (GLDM)<br>Busyness (NGTDM)<br>Complexity (NGTDM)<br>Contrast (NGTDM)<br>Strength (NGTDM)	
**GLCM (4/22)**		
Cluster shade		Independency of voxel values between neighbouring voxels
Correlation		Dependency of voxel values between neighbouring voxels
Inverse measure of correlation 1	Inverse measure of correlation 2 (GLCM)	Complexity of the texture
Inverse variance		Local homogeneity
**GLRLM (1/16)**		
Grey level nonuniformity (GLRLM)	Inverse difference moment normalized (GLCM)<br>Inverse difference normalized (GLCM)<br>Large area high grey level emphasis (GLSZM)<br>Grey level nonuniformity (GLDM)	Similarity of voxel values
**GLSZM (2/16)**		
Low grey level zone emphasis	Small area low grey level emphasis (GLSZM)	Clusters of voxels with low value
Size zone nonuniformity normalized	Small area emphasis (GLSZM)	Variability of cluster sizes
**GLDM (2/14)**		
Large dependence high grey level emphasis		Large clusters of voxels with high values
Small dependence low grey level emphasis		Small clusters of voxels with low values
**NGTDM (0/5)**		
